# The association between serum Sestrin2 and the risk of coronary heart disease in patients with type 2 diabetes mellitus

**DOI:** 10.1186/s12872-022-02727-1

**Published:** 2022-06-21

**Authors:** Xue Tian, Yu Gao, Min Zhong, Mowei Kong, Lihua Zhao, Zengbin Feng, Qitian Sun, Jianqiu He, Xiaoyan Liu

**Affiliations:** 1 Department of Endocrinology, Affiliated Hospital of Chengde Medical University, Chengde, China; 2 Department of Cardiology, Affiliated Hospital of Chengde Medical University, Chengde, China

**Keywords:** Type 2 diabetes mellitus, CHD, Sestrin2, Oxidative stress

## Abstract

**Background:**

Coronary heart disease (CHD) is one of the most common causes of morbidity and mortality in type 2 diabetes mellitus (T2DM). Oxidative stress is one of the important contributors to the pathogenesis of CHD. Sestrin2 is a stress-induced antioxidant protein that plays a important role in T2DM and CHD. However, the relationship between serum Sestrin2 levels and T2DM with CHD remains unclear.

**Aim:**

This study aimed to investigate the relationship between serum Sestrin2 levels and CHD in patients with type 2 diabetes.

**Methods:**

A total of 70 T2DM patients with CHD and 69 T2DM patients were enrolled in this study. Clinical features and metabolic indices were identified. Serum Sestrin2 was measured by ELISA.

**Results:**

Serum Sestrin2 levels in T2DM-CHD groups were significantly lower compared with the T2DM group (11.17 (9.79, 13.14) ng/mL vs 9.46 (8.34, 10.91) ng/mL). Bivariate correlation analysis revealed that serum Sestrin2 levels were negatively correlated with age (r = − 0.256, *P* = 0.002), BMI (r = − 0.206, *P* = 0.015), FBG (r = − 0.261, *P* = 0.002) and Tyg index (r = − 0.207, *P* < 0.014). Binary logistic regression suggested that low serum Sestrin2 levels were related to the increased risk of T2DM-CHD (*P* < 0.05). In addition, the receiver operating characteristic analysis revealed that the area under the curve of Sestrin2 was 0.724 (95% CI 0.641–0.808, *P* < 0.001) to predict T2DM-CHD patients (*P* < 0.001).

**Conclusion:**

The Sestrin2 levels were highly associated with CHD in diabetes patients. Serum Sestrin2 may be involved in the occurrence and development of diabetic with CHD.

## Introduction

In recent decades, Type 2 diabetes mellitus (T2DM) has become one of the most significant health concerns, thus making it a global health priority [1]. It is estimated that by 2040, the world’s prevalence will increase to 629 million [2]. People with T2DM often suffer from multiple complications including macrovascular and microvascular complications [3], it has been known that coronary heart disease (CHD) is one of most common causes of death morbidity for people with diabetes mellitus. CHD accounts for about 65% of the deaths of diabetic patients. However, CHD is a complex condition and the exact cause is not fully understood in diabetic patients.

Oxidative stress, inflammation, endothelial dysfunction, and the release of proinflammatory cytokines are thought to countribute to the pathogenesis of both T2DM and CHD [4]. More evidence suggests that T2DM and CHD are induced by oxidative stress in greater numbers in recent years [5, 6]. Therefore, finding effective antioxidant targets are the focus of the current research.

Sestrin2 is a highly conserved antioxidant protein which was initially identified as hypoxia-induced gene 95 (Hi95) [7, 8]. As a protective factor in physiological and pathological states, Sestrin2 regulates oxidative stress, autophagy, endoplasmic reticulum stress, and inflammation [9]. Sestrin2 inhibits oxidative stress and pro-inflammatory signaling through mechanisms involved with AMPK and mTOR complex 1 (mTORC1), as well as regulating metabolism under stress [10, 11]. In the earliest studies [12, 13], it has been demonstrated that Sestrins play a vital role in the treatment of chronic diseases, such as diabetes, atherosclerosis, cancer, and others. Sestrin2, however, has not yet been tested for its potential role in predicting the risk of CHD with established T2DM.

Therefore, circulating levels of Sestrin2 may provide insight into and induce incident risks related to CHD among diabetic patients with T2DM, thereby presenting new possibilities for preventing CHD-T2DM. Here we compared Sestrin2 serum levels between people with or without CHD, all of whom have T2DM. Besides, the study explores the relationship between CHD risk factors and circulating Sestrin2 concentrations within the T2DM groups specifically.

## Participants and methods

### Study population

A total of 139 patients with T2DM who were diagnosed and treated in our hospital from December 2020 to July 2021 were retrospectively studied. There were 69 patients with T2DM complicated with CHD and 70 patients with T2DM and without CHD complications. All patients agreed to participate in this study and signed an informed consent form. The study was conducted in accordance with the Declaration of Helsinki (as revised in 2013). The inclusion criteria were as follows: (I) patients diagnosed with T2DM according to the criteria of the Chinese Guidelines for Diabetes Prevention and Treatment; and (II) patients with stable CHD, defined as prior myocardial infarction (MI), prior coronary revascularization, or multivessel CHD confirmed by coronary angiography, were eligible. The following patients were excluded from the study: (I)Patients presenting with unstable angina, non-ST-segment elevation MI, or ST-segment elevation MI; (II) patients with severe or active infectious diseases; (III) patients with insufficiencies of the liver and/or kidneys; (IV) patients with malignant tumors; (V) patients with severe hematological diseases; (VI) patients with T1DM and special types of diabetes; and (VII) patients with acute or chronic complications of diabetes.

### Anthropometric evaluation

Clinical and biochemical data were collected using standardised protocols, which were subject to assurance measures and extensive quality control by well‐trained personnel. Baseline characteristics were recorded including age, sex, history of diabetes mellitus, history of smoking, history of hypertension, history of drug. Body mass index (BMI) was calculated as the body weight (kg) divided by the square of the height (m^2^). Blood pressure was measured by trained doctors or nurses using a sphygmomanometer after the patient had rested for at least 10 min and were determined as the mean of 3 measurements taken 1 min apart.

### Blood samples collection and biochemical variable examination

Biochemical parameters such as blood urea nitrogen (BUN), serum creatinine (SCr), fasting blood glucose (FBG), glycosylated hemoglobin A1c (HbA1c), total cholesterol (TC), triglyceride (TG), high-density lipoprotein cholesterol (HDL-C), and low-density lipoprotein cholesterol (LDL-C) were measured using standard procedures in the hospital clinical laboratory. All participants had been fasting for 10 h before blood collection. A volume of 5 mL of cubital venous blood was drawn from each patient and transferred to a vacuum blood collection tube.

### Measuring serum Sestrin2

Blood samples drawn from each participant were centrifuged at 3000 rpm for 15 min, and then the obtained sera were kept at − 80 °C until analysis. Serum levels of Sestrin2 were determined by the enzyme-linked immunosorbent assay (ELISA) technique. The serum concentrations of Sestrin2 were analyzed by Human SESN2 ELISA kits (Fine Biotech, Wuhan, China; catalog number, EH1556). The intra- and interassay coefficients of variation were < 10% and < 12%, respectively.

### Statistical analysis

SPSS 26.0 statistical software was used for the data analysis. The variables in accordance with the normal distribution are represented by mean ± SD, and the variables with skewed distribution are represented by M (Q1, Q3). The counting datas were expressed by the number of cases, and the difference between the two groups was tested by chi-square test. Pearson correlation analysis was used to analyze the correlation between Sestrin2 and other clinical variables. The participants were divided into four groups based on their Sestrin2 quartiles in order to assess the independent impact factors of T2DM-CHD, that is, quartile 1: < 8.74 ng/mL; quartile 2: 8.74–10.18 ng/mL; quartile 3: 10.18–12.37 ng/mL; quartile 4: ⩾ 15.28 ng/mL. To assess the independent impact factors of T2DM-CHD, trend χ2 test and binary logistic regression analysis were performed. The validity of predicting T2DM complicated with CHD by calculating serum Sestrin2 level through the analysis of ROC curve. A value of 95% was used for CI. A two-sided *P* value < 0.05 was considered statistically significant.

## Results

### Baseline clinical characteristics and biochemical measurements

The results obtained from the comparison between clinical and laboratory parameters are given in Table 1. No statistically differences were found in gender, smoking status, SBP, DBP, HbA1c, TyG index, TG and HDL-C, between the two groups. Patients in the T2DM-CHD group had higher BMI, FBG, Cr and BUN, while lower TC and LDL-C compared to with the participants in the control group (*P* < 0.05). The incidences of other conventional risk factors for CHD including hypertension and Diabetic durations were also significantly higher in the T2DM-CHD group (*P* < 0.05).

**Table 1:** Clinical characteristics of the study population

	T2DM	T2DM-CHD	*P* value
Gender (M %)	32 (45.7%)	38 (55.1%)	0.27
Age (years)	55.76 ± 6.55	60.41 ± 6.91	< 0.001
Diabetic durations (years)	5.5 (1, 12)	12 (6, 16)	< 0.001
Hypertension (%)	29 (41.4%)	49 (71.0%)	< 0.001
Smoking status(%)	26 (37.1%)	25 (36.2%)	0.91
BMI (kg/m2)	23.88 ± 2.40	24.72 ± 2.35	0.04
SBP (mmHg)	132 (126, 140)	130 (125, 144)	0.602
DBP (mmHg)	77.5 (68, 90)	80 (70, 85)	0.67
FBG (mmol/L)	8 (6.8, 10.7)	10 (7.6, 12.3)	0.011
HbA1c (%)	9.23 ± 2.06	8.88 ± 1.93	0.303
TyG index	9.56 ± 0.70	9.56 ± 0.67	0.989
TC(mmol/L)	4.92 ± 1.20	4.11 ± 1.21	< 0.001
TG (mmol/L)	2.21 (1.43, 3.19)	2.03 (1.21, 2.89)	0.216
HDL-C(mmol/L)	1.03 (0.85, 1.19)	0.95 (0.83, 1.17)	0.281
LDL-C(mmol/L)	2.77 (1.95, 3.48)	2.0 (1.53, 2.91)	0.001
Cr (umol/L)	55.40 (45.00, 65.10)	67.50 (52.10, 79.20)	0.001
BUN (nmol/L)	5.58 (4.58, 6.49)	5.79 (5.04, 7.69)	0.024
Statins (%)	6 (8.6%)	40 (58.0%)	< 0.001

Data are presented as the mean value ± SD or median (interquartile range)

BMI, body mass index; SBP, systolic blood pressure; DBP, diastolic blood pressure; FBG, fasting blood glucose; HbA1c, hemoglobin a1c; TyG index, Triglyceride glucose TyG index; TC, total cholesterol; TG, triglycerides; HDL-C, high-density lipoprotein cholesterol; LDL-C, low-density lipoprotein cholesterol; Cr, creatinine; BUN, blood urea nitrogen

*P* < 0.05 was considered statistically significant

**Fig. 1:**
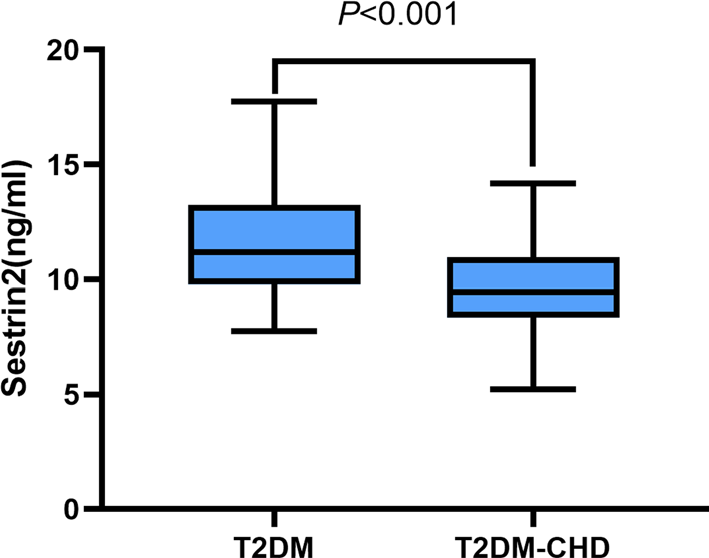
Serum Sestrin2 levels in patients with T2DM and T2DM-CHD groups. *P* value < 0.05 was considered significant

As illustrated in Fig. 1, Sestrin2 demonstrated significant differences in plasma samples between T2DM and T2DM-CHD. The levels of circulating Sestrin2 were significantly lower in T2DM-CHD patients than in T2DM subjects (9.46(8.34,10.91)ng/mL vs 11.17 (9.79,13.14)ng/mL; *P* < 0.001).

### Correlation between Sestrin2 and other variables

**Fig. 2:**
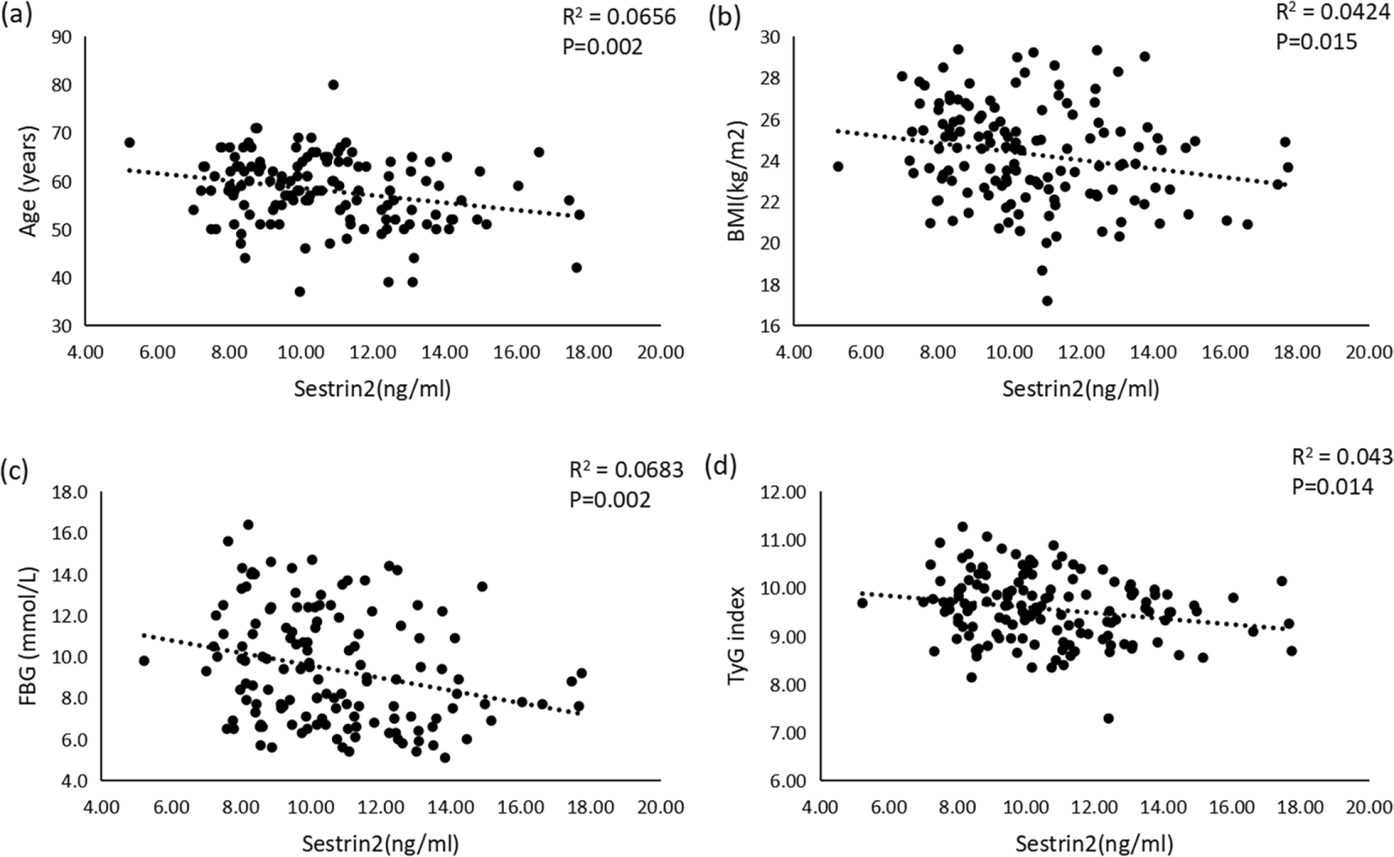
Correlation of the serum Sestrin2 levels and the clinical indexes. The serum Sestrin2 levels in the patients were negatively correlated with the **a** Age, **b** BMI, **c** FBG, and **d** Tyg index. Each symbol represents an individual patient. The correlations were evaluated by performing Pearson correlation test. *P* < 0.05, indicated a statistically significant difference

According to Pearson correlation analysis, Sestrin2 levels were negatively correlated with age (r = − 0.256, *P* = 0.002), BMI (r = − 0.206, *P* = 0.015), FBG (r = − 0.261, *P* = 0.002) and Tyg index (r = − 0.207, *P* < 0.014) (Fig. 2). However, Sestrin2 levels were not associated with SBP, DBP, HbA1c, TG, LDL-c, and other variables (Table 2).

**Table 2:** Simple correlation analysis of serum Sestrin2 and parameters

Parameters	Correlation coefficient (r)	*P* value
Age	− 0.256	0.002
BMI	− 0.206	0.015
SBP	− 0.043	0.617
DBP	− 0.034	0.69
FBG	− 0.261	0.002
HbA1c	0.092	0.28
TyG index	− 0.207	0.014
TG	− 0.138	0.105
HDL-C	0.017	0.846
LDL-C	0.138	0.105
Cr	− 0.106	0.215
BUN	− 0.137	0.109

BMI, body mass index; SBP, systolic blood pressure; DBP, diastolic blood pressure; FBG, fasting blood glucose; HbA1c, hemoglobin a1c; TyG index, Triglyceride glucose TyG index; TG, triglycerides; HDL-C, high-density lipoprotein cholesterol; LDL-C, low-density lipoprotein cholesterol; Cr, creatinine; BUN, blood urea nitrogen

*P* < 0.05 was considered statistically significant

### The association between Sestrin2 and T2DM-CHD

From the quartile 1 to the quartile 4 of Sestrin2, the percentage of T2DM-CHD was 37.7%, 27.5%, 24.6% and 10.1%, respectively. The result indicated that with the increase of Sestrin2 levels in the population, the percentage of T2DM-CHD presented a general downward trend. The trend χ2 test showed that the difference was statistically significant (*P* < 0.001).

Binary logistic regression analyses indicated that Sestrin2 levels were negatively associated with T2DM-CHD, as Sestrin2 increased, the prevalence of CHD decreased after adjusting for confounding variables (*P* < 0.05). Taken Sestrin2 as classification variable (quartile 1–4) for the regression model, we adjusted for confounding variables before the bottom quartile of Sestrin2 compared with the top, an OR of 9.167(95% CI 2.003–41.954, *P* < 0.05) was observed (Table 3).

**Table 3:** Binary multivariate stepwise logistic regression of the independent factors for T2DM (Sestrin2 as continuous variable or categorical variable)

Variable	Model 1	Model 2	Model 3
sestrin2	0.624 (0.496, 0.784)*	0.666 (0.518, 0.856)*	0.66 (0.508, 0.857)*
sestrin2 quartile 4	Ref	Ref	Ref
sestrin2 quartile 3	13.339 (1.06, 12.528)*	2.862 (0.769, 10.649)	2.524 (0.621, 10.264)
sestrin2 quartile 2	4.782 (1.403, 16.295)*	3.172 (0.819, 12.289)	3.48 (0.874, 13.866)
sestrin2 quartile 1	0.075 (3.505, 50.765)*	8.737 (2.046, 37.304)*	9.167 (2.003, 41.954)*

Data were odds ratio (95% confidence interval), which were calculated by logistic analyses. Model 1 was adjusted for age, sex, hypertension, diabetic durations and smoking status; Model 2 was adjusted for Model 1, BMI, FBG, HbA1c and TyG index; Model 3 was adjusted for Model 2, TC, TG, HDL-C, LDL-C, Scr and BUN

*P < 0.05

### The ROC curve analysis

**Fig. 3:**
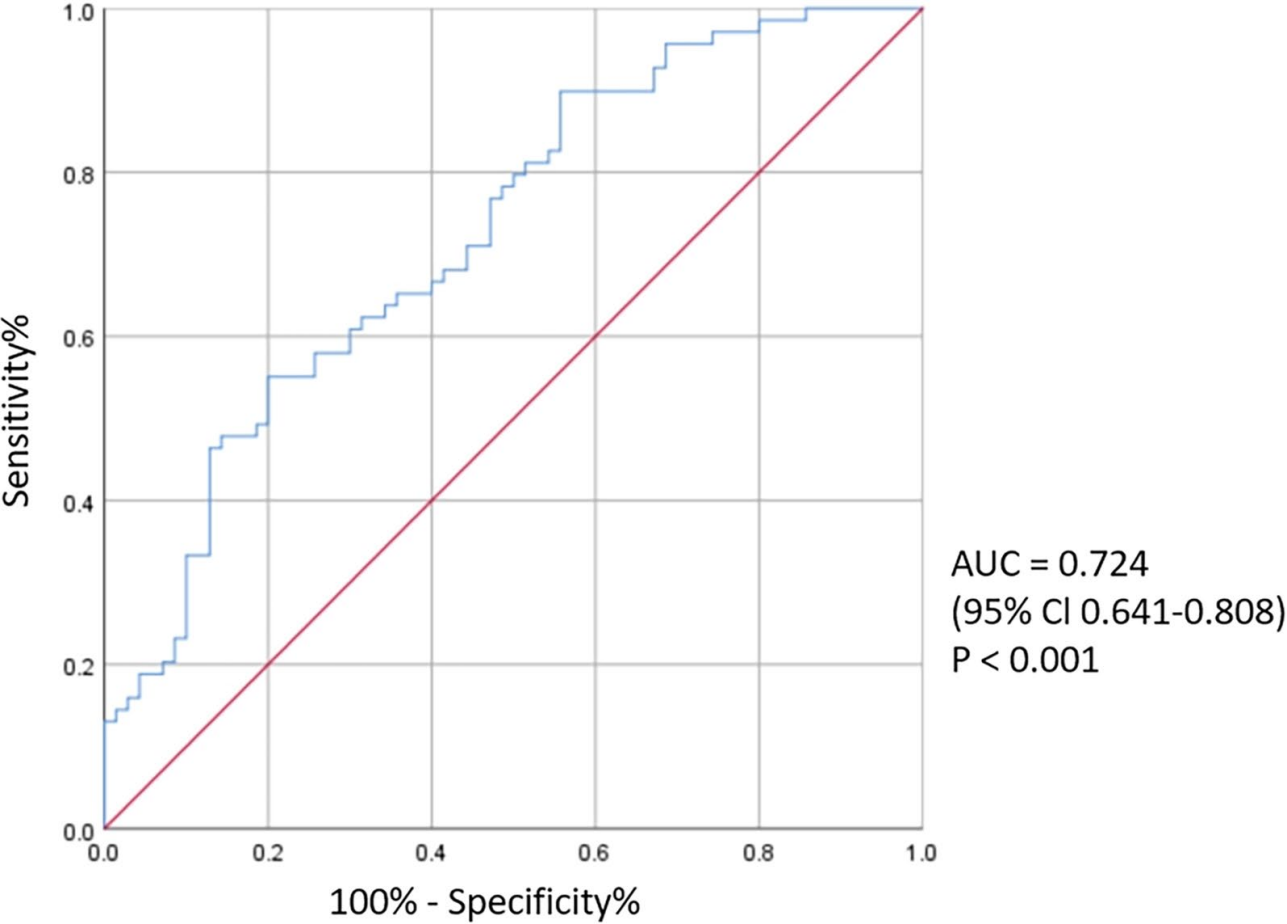
Receiver operating characteristic (ROC) curve analyse was carried out for diagnose CHD in T2DM patients according to the Sestrin2 levels. The comparison of the area under the curve (AUC) was performed by a *P* value < 0.05

The ROC curve for circulating Sestrin2 levels to predict T2DM-CHD is shown in Fig. 3. Based on ROC analysis, the best cutoff value of circulating levels of Sestrin2 was calculated to be 9.6 ng/mL with a sensitivity of 55.1%, a specificity of 80%, and an under curve area of 0.724 (95% CI 0.641–0.808, *P* < 0.001) for T2DM-CHD prediction.

## Discussion

In this study, we found that the serum Sestrin2 levels in T2DM with CHD were lower than that in T2DM patients. This study found that serum Sestrin2 levels were significantly negatively correlated with the age, BMI, FBG and Tyg index in T2DM patients. In addition, low levels of Sestrin2 are a risk factor for CHD in T2DM patients.

T2DM is a metabolic disorder characterized by chronic hyperglycemia and improper lipid, carbohydrate, and protein metabolism, caused by insulin resistance and inadequate insulin release [14]. Patients with T2DM are at a higher risk of cardiovascular diseases, especially CHD, due to their dysregulated metabolism of lipids [15]. In spite of the importance of all complications associated with type 2 diabetes, CHD continues to be the leading cause of morbidity and mortality for this population [16]. In light of these findings, we must refocus on aggressive risk reduction of cardiovascular disease for patients living with type 2 diabetes, especially those who have already been diagnosed with CHD. Research studies have demonstrated that the production of the Reactive oxygen species (ROS) may play a significant role in heart disease [17, 18]. A key regulator of cellular physiological metabolism, ROS, is mainly produced in mitochondria [19]. Researchers have demonstrated that ROS, as an intracellular signal molecule, influences a variety of physiological processes in cardiomyocytes [20]. In addition, cardiovascular diseases are widely reported to be associated with impaired autophagy processes, resulting in the death of cardiomyocytes as the damaged autophagosomes are not cleared properly [21, 22]. It has been demonstrated that Sestrin2 exerts its antioxidant activity by reducing the production of ROS and by stimulating autophagy as an oxidative stress-induced protein regulated by p53 [23].

Researchers discovered that the N-terminal domain of Sestrin2 shared allosteric properties with alkyl hydroperoxide reductase D from Mycobacterium tuberculosis, and they acted as antioxidants [8]. The ability to induce sulfiredoxin expression later was shown to be due to Sestrin2 acted as a peroxidase reductase [24]. Sestrin2 is a direct inhibitor, but it is also capable of regulating signaling pathways to exert antioxidant effects. By enhancing autophagy-dependent degradation of kelch-like ECH-related protein 1 (keap1), sestrin2 can induce the expression of transcription factor Nrf-2 that in combination with the antioxidant response element (ARE) exerts cell protection functions [25]. Sestrin2 can also inhibit ROS accumulation by inhibiting the mTOR and DADPH-dependent oxidase 4 signaling pathways [26], as well as by enhancing mitochondrial phagocytosis [27]. Using plasma Sestrin2 levels as biomarkers, Xiao et al. [28] noticed a positive correlation between Sestrin2 levels and malondialdehyde and a negative correlation with superoxide dismutase in patients with aortic dissection, which is evidence of Sestrin2’s antioxidant activity.

According to recent studies [29–31], Sestrin2 plays a crucial role in metabolic disorders of cardiovascular events, such as oxidative stress, AMPK/mTOR ignalling pathway, and Nrf2-induced autophagy. In truth, there is a controversy regarding the relationship between Sestrin2 deficiency and CHD. According to our current data, serum Sestrin2 concentration was found to be significantly lower in T2DM patients with CHD as compared with T2DM patients without CHD. Despite the fact that this is the first study examining the association between Sestrin2 and T2DM-CHD, the possible mechanism of association between low levels of Sestrin2 and T2DM-CHD remains unclear. This may be due to the compensatory mechanism of Sestrin2 may be insufficient to regulate abnormal metabolism. As the Sestrin2 level decreases, ROS will accumulate and autophagy will be inhibited, leading to CHD. It is suggested that the low Sestrin2 is a risk factor for T2DM-CHD. In the next step of our research, we will explore this possibility. Sundararajan et al. [32] measured the plasma Sestrin2 levels in individuals with normal glucose tolerance (NGT) (n = 46), dyslipidemia (n = 42), and patients with Type 2 diabetes with (n = 41) and without dyslipidemia (n = 40). A significant negative correlation was shown between Sestrin2 and atherogenic risk factors, as well as severity of atherogenic index. The findings of their study are similar to ours. However, the conclusion of this study is not in agreement with the conclusions of other studies. Ye et al. [33] reported that the Sestrin2 levels in patients with coronary artery disease (CAD) were higher than those in those without CAD. Sestrin2 levels among patients with unstable angina (UA) or acute MI (AMI) were much higher than those in patients with stable angina (SA). In another study [34], Sestrin2 was measured in plasma in 304 patients who underwent coronary angiography for suspected CAD. Significantly higher plasma concentrations of Sestrin2 were seen in the CAD patients compared with the non-CAD patients. Sestrin2 levels also increased in a stepwise manner that was dependent on the severity of CAD (defined as the number of > 50% stenotic vessels), and they were highest in patients with severe CAD. Indeed, the current work couldn’t explain this contradiction. These may be due to the increased plasma Sestrin2 levels may be a compensate response. As the disease advances, the compensatory mechanism of Sestrin2 is no longer sufficient to control the intracellular environment, which further decreases as time progresses.

Diabetes and cardiovascular disease are both characterized by obesity as one of their leading causes [35]. Impairing glucose tolerance is an important factor influencing progression of atherosclerosis, according to previous studies [36]. In our study, we found that serum Sestrin2 levels were negatively correlated with the BMI, FBG and Tyg index in patients with T2DM, indicating that serum Sestrin2 may be involved in the occurrence and development of T2DM-CHD. As a result, the result of the study was consistent with that of the previous one [34]. As well, Nourbakhsh et al. [37] found that the serum levels of Sestrin2 were negatively correlated with BMI, HbA1c, serum creatinine, glucose, and albumin in patients with T2DM. Further, they found lower levels of Sestrin2 in obese children and obese children with diabetes when compared to children with normal weight, and these levels were negatively correlated with BMI, HbA1c, and glucose levels [38]. On contrary, the study by Chung et al. [39] found that serum Sestrin2 levels were higher in obese and T2DM patients when compared to the healthy control group. These levels were positively correlated with BMI, levels of serum triacylglycerol, glucose, C-reactive protein and the degree of insulin resistance. The present study couldn’t explain this inconsistency.

Another novel finding, in our present study, serum Sestrin2 levels showed about 72.4% AUC to differentiate patients with T2DM-CHD from diabetic only cases. The sensitivity was 55.1% and specificity was 80%. Moreover, we found that the risk of T2DM-CHD gradually decreased across increasing quartiles of Sestrin2. Meanwhile, our findings showed that low serum Sestrin2 levels were strongly associated with T2DM-CHD prevalence, even after adjustment for a variety of risk factors, supporting the hypothesis that Sestrin2 deficiency was a risk factor for this disease.

There are a number of limitations inherent in the current study. Firstly, the sample size was quite small as it was a single-center study. All the participants were from the same region. Consequently, it was impossible to exclude patients’ selection bias. Secondly, in our study, we found that the TC and LDL-C level in T2DM with CHD was lower than that in T2DM patients, as all were being administered statins in accordance to their risk profiles. There was no discussion in this study about how lipid-lowering agents affect serum Sestrin2, which might affect the results of the experiment. Additionally, control subjects with T2DM could not be excluded from confounding effects such as those caused by the diseases themselves or other medicine treatments. Finally, this was only an observational study, and causality could not be established in this case.

## Conclusions

In conclusion, According to our findings, the serum Sestrin2 levels in type 2 diabetes are associated with CHD, and age, body mass index, FBG, and Tyg index are negatively correlated with the Sestrin2 levels. Based on these results, Sestrin2 could play a significant role in the process of developing T2DM with CHD and be a risk factor. Despite the fact that the causal relationship between Sestrin2 and T2DM-CHD is inconclusive from the present research results, Sestrin2 levels provide a valuable clinical and reference value in diagnosed patients with T2DM that is complicated by CHD. However, futher study of the specific molecular mechanisms in disease and an expansion of the clinical sample size would be necessary to determine the clinical utility of Sestrin2.

## Data Availability

The data-sets used and/or analyzed during the current study available from the corresponding author on reasonable request.

## Abbreviations

BMI: Body mass index
SBP: Systolic blood pressure
DBP: Diastolic blood pressure
FBG: Fasting blood glucose
HbA1c: Hemoglobin a1c
TyG index: Triglyceride glucose TyG index
TC: Total cholesterol
TG: Triglycerides
HDL-C: High-density lipoprotein cholesterol
LDL-C: Low-density lipoprotein cholesterol
Cr: Creatinine
BUN: Blood urea nitrogen
CHD: Coronary heart disease
T2DM: Type 2 diabetes mellitus

## References

[1] Zhang YB, Pan XF, Chen JX, Xia L, Cao AL, Zhang YG, Wang J, Li HQ, Yang K, Guo KQ, He MA, Pan A (2020). Combined lifestyle factors and risk of incident type 2 diabetes and prognosis among individuals with type 2 diabetes: a systematic review and meta-analysis of prospective cohort studies. Diabetologia.

[2] Sebastiani G, Nigi L, Grieco GE, Mancarella F, Ventriglia G, Dotta F (2017). Circulating microRNAs and diabetes mellitus: a novel tool for disease prediction, diagnosis, and staging?. J Endocrinol Invest.

[3] Elisa DC, Antonio C, Lars R, Marc F, Tina BH, Oliver S, Eberhard S, Joline WJB (2019). Diabetes as a cardiovascular risk factor: an overview of global trends of macro and micro vascular complications. Eur J Prev Cardiol.

[4] Sotirios T, Alexios SA, Evangelos O, George AP, Georgia V, Spyridon P, Spyros D, Dimitris T (2019). The role of inflammation in diabetes: current concepts and future perspectives. Eur Cardiol.

[5] Leonie SR, Lara U, René L, Ronald R (2017). Oxidative stress: promoter of allergic sensitization to protease allergens?. Int J Mol Sci.

[6] Azizi S, Mameghani ME, Mobasseri M, Karamzad N, Mahdavi R (2021). Oxidative stress and nitrate/nitrite (NOx) status following citrulline supplementation in type 2 diabetes: a randomised, double-blind, placebo-controlled trial. J Hum Nutr Diet.

[7] Andrei VB, Tzipora S, Alexander F, Elena Z, Iris K, Hagar K, Svetlana G, Alla F, Ayelet C, Paz E, Rami S, Andrei VG, Peter MC, Elena F (2002). Identification of a novel stress-responsive gene Hi95 involved in regulation of cell viability. Oncogene.

[8] Andrei VB, Anna AS, Elena F, Eugene VK, Peter MC (2004). Regeneration of peroxiredoxins by p53-regulated sestrins, homologs of bacterial AhpD. Science.

[9] Wang LX, Zhu XM, Yao YM (2019). Sestrin 2: Its potential role and regulatory mechanism in host immune response in diseases. Front Immunol.

[10] Sun YF, Wu YW, Tang ST, Liu H, Jiang YP (2020). Sestrin proteins in cardiovascular disease. Clin Chim Acta.

[11] Mazhar P, Ali HE, Assaad AE, Yves G, Shankar M (2017). Sestrin2 as a novel biomarker and therapeutic target for various diseases. Oxid Med Cell Longev.

[12] Jun HL, Andrei VB, Michael K (2013). Sestrins orchestrate cellular metabolism to attenuate aging. Cell Metab.

[13] Wang ML, Xu Y, Liu JF, Ye J, Yuan WH, Jiang HM, Wang Z, Jiang H, Wan J (2017). Recent insights into the biological functions of sestrins in health and disease. Cell Physiol Biochem.

[14] Wang S, Yong H, He XD (2021). Multiomics: opportunities for research on mechanism of type 2 diabetes mellitus. World J Diabetes.

[15] Iciar MT, Cristina SC, Amparo SG, Francisco JDCG (2014). Type 2 diabetes and cardiovascular disease: have all risk factors the same strength?. World J Diabetes.

[16] Matthew AC, Ph GS, Sidney CS, Kim E, Ohman EM, Shinya G, Julia K, Kyungah I, Peter WFW, Deepak LB, REACH Registry Investigators (2015). Impact of diabetes mellitus on hospitalization for heart failure, cardiovascular events, and death: outcomes at 4 years from the Reduction of Atherothrombosis for Continued Health (REACH) Registry. Circulation.

[17] Zhang PY, Xu X, Li XC (2014). Cardiovascular diseases: oxidative damage and anti-oxidant protection. Eur Rev Med Pharmacol Sci.

[18] Gao AB, Francisco SC, Chen X, Jing Y, Li W, Peng TH, Lv YC (2017). Implications of sortilin in lipid metabolism and lipid disorder diseases. DNA Cell Biol.

[19] Gao AB, Jiang JY, Xie F, Chen LX (2020). Bnip3 in mitophagy: novel insights and potential therapeutic target for diseases of secondary mitochondrial dysfunction. Clin Chim Acta.

[20] Yao RQ, Ren C, Xia ZF, Yao YM (2021). Organelle-specific autophagy in inflammatory diseases: a potential therapeutic target underlying the quality control of multiple organelles. Autophagy.

[21] Quan NH, Sun WQ, Wang L, Chen X, Bogan JS, Zhou XC, Cates C (2017). Liu Quan, Zheng Y, Li J, Sestrin2 prevents age-related intolerance to ischemia and reperfusion injury by modulating substrate metabolism. FASEB J.

[22] Li YB, Liang PF, Jiang BM, Tang YT, Liu XY, Liu MD, Hui S, Chen C, Hao H, Liu ZG, Xiao XZ (2020). Card9 promotes autophagy in cardiomyocytes in myocardial ischemia/reperfusion injury via interacting with rubicon directly. Basic Res Cardiol.

[23] Silke E, Nathalie D, Bernhard B (2009). Role of sestrin2 in peroxide signaling in macrophages. FEBS Lett.

[24] Hyun AW, Soo HB, Sunjoo P, Sue GR (2009). Sestrin 2 is not a reductase for cysteine sulfinic acid of peroxiredoxins. Antioxid Redox Signal.

[25] Chen MJ, Xi YM, Chen K, Xiao P, Li SJ, Sun XC, Han ZY (2021). Upregulation Sestrin2 protects against hydrogen peroxide-induced oxidative damage bovine mammary epithelial cells via a Keap1- Nrf2/ARE pathway. J Cell Physiol.

[26] Hwang CY, Han YH, Lee SM, Cho SM, Yu DY, Kwon KS (2020). Sestrin2 attenuates cellular senescence by inhibiting NADPH oxidase 4 expression. Ann Geriatr Med Res.

[27] Kim MJ, Bae SH, Ryu JC, Kwon Y, Oh JH, Kwon J, Moon JS, Kim K, Miyawaki A, Lee MG, Shin J, Kim YS, Kim CH, Ryter SW, Choi AMK, Rhee SG, Ryu JH, Yoon JH (2016). SESN2/sestrin2 suppresses sepsis by inducing mitophagy and inhibiting NLRP3 activation in macrophages. Autophagy.

[28] Xiao T, Le Z, Huang Y, Shi Y, Wang J, Ji QW, Ye J, Lin YZ, Liu HT (2019). Sestrin2 increases in aortas and plasma from aortic dissection patients and alleviates angiotensin II-induced smooth muscle cell apoptosis via the Nrf2 pathway. Life Sci.

[29] Wang ZW, Bu L, Peng Y, Feng SJ, Xu F (2019). Alleviation of sepsisinduced cardiac dysfunction by overexpression of sestrin2 is associated with inhibition of ps6k and activation of the pampk pathway. Mol Med Rep.

[30] Zhang LL, Zhang ZJ (2018). Sestrin2 aggravates oxidative stress of neurons by decreasing the expression of nrf2. Eur Rev Med Pharmacol Sci.

[31] Quan N, Wang L, Chen X, Luckett C, Cates C, Rousselle T, Zheng Y, Li J (2018). Sestrin2 prevents age-related intolerance to post myocardial infarction via ampk/pgc-1alpha pathway. J Mol Cell Cardiol.

[32] Sundararajan S, Jayachandran I, Subramanian SC, Anjana RM, Balasubramanyam M, Mohan V, Venkatesan B, Manickam N (2021). Decreased Sestrin levels in patients with type 2 diabetes and dyslipidemia and their association with the severity of atherogenic index. J Endocrinol Invest.

[33] Ye J, Wang ML, Xu Y, Liu JF, Jiang HM, Wang Z, Lin YZ, Wan J (2017). Sestrins increase in patients with coronary artery disease and associate with the severity of coronary stenosis. Clin Chim Acta.

[34] Yoshimi K, Masayuki A, Emi S, Yukinori I, Reiko O, Kazuo K, Yukihiko M (2020). Association between plasma sestrin2 levels and the presence and severity of coronary artery disease. Dis Markers.

[35] Shivani AP, Mohan D, Roopa S, Mohammed KA, Deksha K, Ruby G, Dorothy L, Nikhil T, Viswanathan M, Kadir MM, Zafar F, Dorairaj P, Narayan KMV (2017). Comparison of multiple obesity indices for cardiovascular disease risk classification in South Asian adults: the CARRS Study. PLoS ONE.

[36] Roberto S, Philippe G, Antonio G, Antonino DP, Agata MR, Francesco P, Philippe C, Alban R, Eric B, David R (2016). HbA1c increase is associated with higher coronary and peripheral atherosclerotic burden in non diabetic patients. Atherosclerosis.

[37] Khalid MM, Osamah AR (2020). Association of serum sestrin 2 and betatrophin with serum neutrophil gelatinase associated lipocalin levels in type 2 diabetic patients with diabetic nephropathy. J Diabetes Metab Disord.

[38] Khalid MM, Osamah AR, Osama AW, Abdullah AN (2021). Investigation of the levels of circulating miR-29a, miR-122, sestrin 2 and inflammatory markers in obese children with/without type 2 diabetes: a case control study. BMC Endocr Disord.

[39] Chung HS, Hwang HJ, Hwang SY, Kim NH, Seo JA, Kim SG, Kim NH, Baik SH, Choi KM, Yoo HJ (2018). Association of serum Sestrin2 level with metabolic risk factors in newly diagnosed drug-naïve type 2 diabetes. Diabetes Res Clin Pract.

